# Feeding habit and diet composition of three fish species inhabiting Sor River, Baro-Akobo Basin of Ethiopia, East Africa

**DOI:** 10.1371/journal.pone.0319927

**Published:** 2025-03-21

**Authors:** Tujuba Ayele Tesso, Limatu Kebede Nurgi

**Affiliations:** Department of Biology, College of Natural and Computational Sciences, Mattu University, Mettu, Ethiopia; University of Kalyani, INDIA

## Abstract

The purpose of this study was to investigate feeding habit and diet composition of three fish species *(Oreochromis niloticus, Labeo forskalii* and *Labeobarbus intermedius*) inhabiting Sor River, Baro-Akobo basin of Ethiopia. Stomach contents of these fish species were examined. A total of 73 fish gut samples were collected from fishermen on site from February to March 2022. The diet composition of each species was analysed and expressed as percentage of frequency of occurrence (% F), numerical occurrence and gravimetric composition (% G). The major food items in the stomach of each species were determined using percentage index of relative importance (% IRI). The study showed that *O. niloticus* from Sor River feeds on phytoplankton, macrophytes and detritus as major prey. The stomach of *L. forskalii* was found to have Phytoplankton, macrophytes, insects, fish larvae, detritus and others. The gut content analysis of *L. intermedius* revealed phytoplankton and detritus found to be the dominant food constituents with % IRI 23.72% and 28.04% respectively. Fish larvae and flatworms are important diet constituents. The diet breadth index which measures the diversity of prey items consumed by each fish species was 0.43 for *O. niloticus,* 0.61 for *L. intermedius* and 0.64 for *L. forskalii,* respectively. According to their diet breadth and overlap indices, the three fish species had an omnivorous feeding habit which is useful to enhance fisheries and aquaculture development in the study area.

## Introduction

Ethiopian water bodies, lakes and rivers are rich in commercially important fish species. Particularly, Southwest Ethiopia is endowed with inland water bodies which are rich in faunal diversity such as fish, amphibians and reptiles. Riverine fishing activity and marketing systems of Baro-Akobo Basin was roughly assessed by Abegaz *et al* [1] and other NGOs. Study by Melaku *et al* [2] identified nine fish species from Sor and Geba rivers which are economically important. *Labeo forskalii, Labeobarbus intermedius, and Oreochromis niloticus* are common fish species inhabiting Sor and Gebba rivers [3]. *Oreochromis niloticus and Labeobarbus intermedius* are widely distributed in Ethiopian water bodies [4] and commercially important fish species. The fishing activity, fish production and demand outlook in the riverine systems of Saki, Sese, Dogi, Ganji, Sor, Gabba, Dabana and Dedesa have also been studied [3].

The study of food and feeding habits of freshwater fish species is a subject of continuous research. This is because it makes up a basis for the development of a successful management program on fish capture and culture [5]. Moreover, studies on the natural feeding of fish enable one to identify the trophic relationships present in aquatic ecosystems, identifying feeding composition, structure and stability of food webs in the ecosystem [6,7]. However, feeding habits and diet composition of the major fish species of Sor River have not been studied. Therefore, this study aimed to investigate feeding habits and diet composition of three important fish species inhabiting Sor River, Baro-Akobo basin of Ethiopia, East Africa.

## Materials and methods

### Study area description

**Fig 1:**
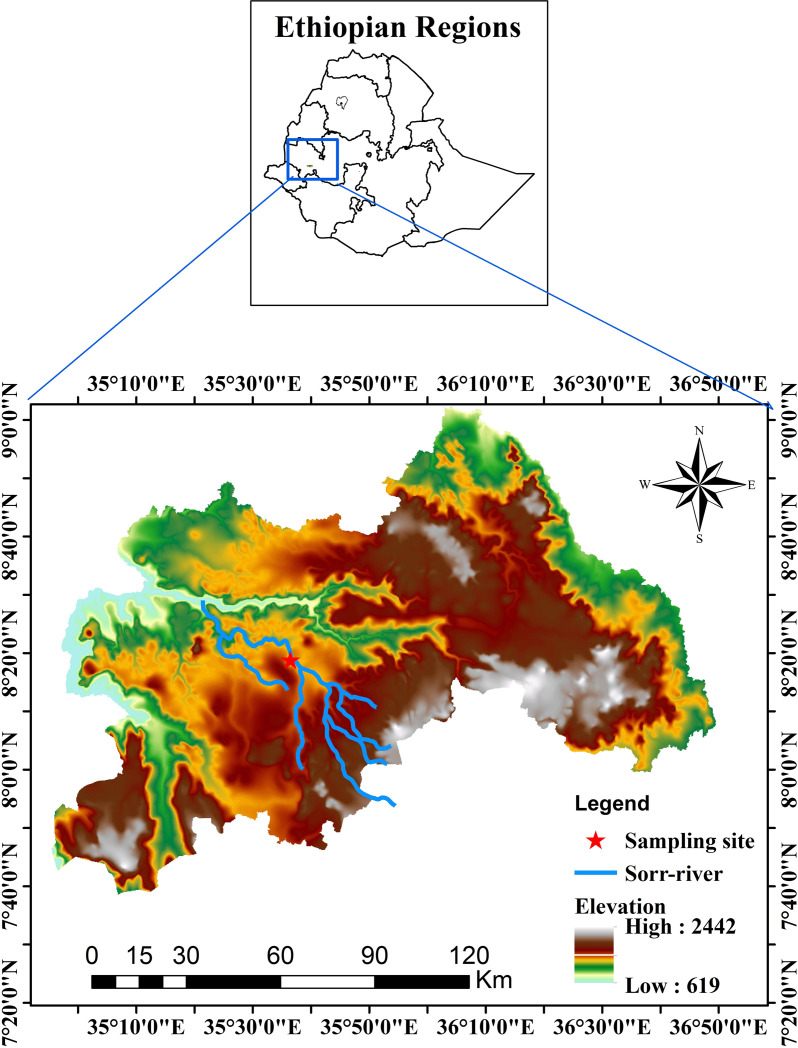
Map of the study area (*Source*: Ethiopian Geospatial information Agency and United States Geological Survey, 2024).

The study was conducted in Mattu district, Ilu Abba Bor zone, Oromia Regional State South-western Ethiopia (Fig 1). Mattu district is one of the eleven districts located along the Sor River some 600 km of Addis Ababa [8]. The Sor River, a prominent river in Mattu District, begins in Sayo, which is a part of the Baro-Akobo basin. It is a tributary of the Geba River on its left. The entire course of the Sor River is lined with a thick forest of Yayo ecosystem. The river is reported to have a pH of 6.9 and a dissolved oxygen content of 4.2 mg/L [8].

### Sample collection and identification

Fish samples were collected from local fishermen of Sor River on site from February to March 2022. Fish sampling consent was obtained from Ilu Aba Bor Zone livestock and fishery institute. Fish were captured by gill nets which were set overnight and collected on the following day morning. The contents of all non-empty guts were collected and preserved in a 5% formalin solution for dietary analysis in the laboratory [9]. Gut contents were collected from the stomach of *O. niloticus* and the anterior portion of the intestine of *L. intermedius and Labeo forskalii.* Food items were identified using dissecting and compound microscopes at Mattu University Biological Science laboratory. Total length (TL) and weight (W) measurements were taken onsite for all specimens collected (n =  31 for *O. niloticus;* n =  28 for *L. intermedius* and n = 14 for *L. forskalii).* Total length was measured from the tip of the snout to the extended tip of the caudal fin to the nearest 0.1 cm, and W was measured to the nearest 0.1 g. The phytoplankton in the stomach contents were counted by using the procedures outlined in Willén [9]. The food items were identified to the possible taxonomic group using descriptions, illustrations and keys in the literature [10–12].

## Data analysis

### Index of relative importance (IRI)

Index of Relative Importance (IRI) was calculated according to Pennak *et al*. [13] as follows:


$$IRI=\%F\left(\%N+\%G\right)$$ (1)

where: % F =  frequency of occurrence of the food item; %N =  numerical percentage of a food item in the stomachs; and % G =  percentage by weight of the food item in the stomach.

### Frequency of occurrence (% F)


$$\text{\% }F=\frac{Ni}{N}*100$$ (2)

Where: % F is the frequency of occurrence of given food I, N_*i*_ is the number of stomachs containing prey I, N is the total number of stomachs with some food.

**Number method:** The number method is based on the counts of food items in the gut content. The number of individuals of each food category in each stomach are recorded & expressed as a percentage of the total number of food items in all fish stomachs examined or as a proportion of the food items of each stomach of Fish examined, which raised to the total percentage composition [14, 15].

### Percentage by number


$$\text{\% N}=\frac{Ni}{Nt}*100$$ (3)

Where: % N is the percentage of food item i; Ni is the number of food item i

Nt is the total number of food (gut content) items

### Percentage weight (G %) of food item


$$\text{\% G}=\frac{Wi}{Wt}*100$$ (4)

Where: % G is the percentage weight of food item i; W*i* is the weight of food item i; Wt is the total weight of food (gut content) items

**Index of stomach fullness (ISF):** Index of stomach fullness expresses the ratio of food weight to body weight. This index is extensively employed & it could be applied to the food in the stomach or to that in the whole digestive tract & calculated using the formula as follows: -

### Index of stomach fullness (ISF)


$$ISF=\frac{Wg}{Wf}*100$$ (5)

Where: % Wg is the weight of the stomach contents (g); Wf is fish body weight

The diversity of prey items consumed by each fish species was measured using a diet breadth index (B), which was calculated as follows:


$$B=\frac{1}{\sum_{i=1}^{n}Pi^{2}}$$

and standardize it (B_A_) to a scale from 0 to 1.0 as:

Where Pi is the proportion of each prey category, n =  the number of different prey Categories. B was standardized according to Gelwick and Mathews [16] to scale from 0 to 1 as follows:$BA=\frac{B-1}{n-1}$

BA can be used as a measure of prey diversity eaten by each fish species (or size class) in a single habitat type or in multiple segments of a stream.

The extent of diet overlap between the two fish species was assessed using Horn’s index (H) as:


$$H=\frac{2\sum_{i=1}^{n}\text{Pij Pik}}{\left(\frac{\sum_{i=1}^{n}{\text{Pij}}^{2}}{{\text{J}}^{2}}+\frac{\sum_{i=1}^{n}{\text{PiK}}^{2}}{{\text{K}}^{2}}\right)\text{JK}}$$

where

Pij = volume proportion of prey i in the total preys consumed by fish species j

Pik =  volume proportion of prey i in the total preys consumed by fish species k

n =  the total number of prey categories J, K =  total amount (mg) of all the preys consumed by fish species j and k respectively [16].

## Results

Sor River is a tributary of the Baro-Akobo basin that contains a variety of fish species. A total of nine fish species were reported from Sor and Gaba Rivers in an earlier study [2]. The stomach contents of three fish were examined in the current study. The total length, weight, and stomach fullness of these fish were all measured (Table 1).

**Table 1:** Average length, weight and index of stomach fullness of *Labeo forskalii* (n = 14, *Labeobarbus intermedius* (n = 28) and *Oreochromis niloticus* (n = 31) from Sor River.

Fish species	Mean L (±SD)	Mean Wg	ISF
*Labeo forskalii (n = 14)*	26.79 ± 5.63	414.73 ± 146.15	24.12 ± 5.13
*Labeobarbus intermedius (n = 28)*	29.11 ± 7.41	388.32 ± 132.04	16.83 ± 6.05
*Oreochromis niloticus (n = 31)*	19.35 ± 5.12	143.66 ± 7.13	20.43 ± 8.14

n = number of fish samples; SD=standard deviation; ISF=Index of Stomach Fullness.

### Feeding habit and diet composition

Three fish species samples; *Oreochromis niloticus, Labeo forskalii, and Labeobarbus intermedius* were collected and analysed for their stomach content from Sor River. Frequency of occurrence (% F), percentage by number (% N), percentage by weight (G %) and Index of relative importance (IRI %) of the food items were analysed.

### Nile tilapia (*Oreochromis niloticus*)

**Fig 2:**
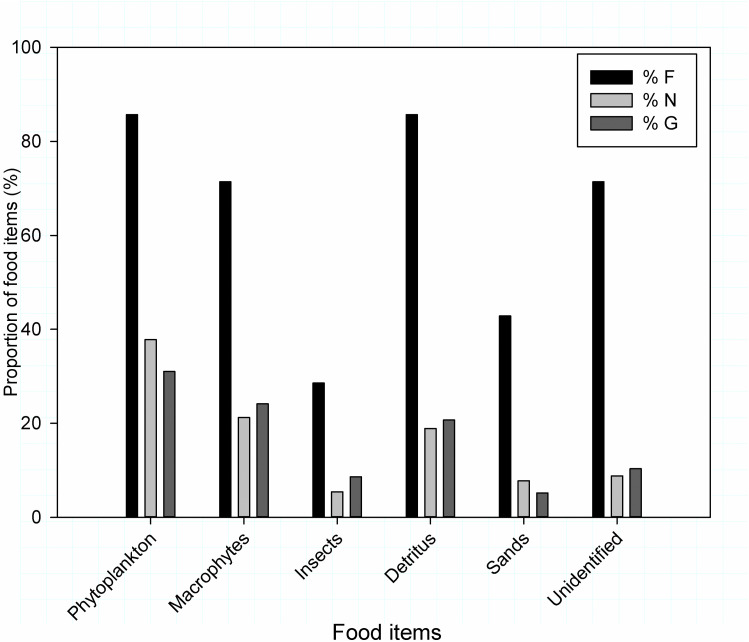
The percentage contribution of food items consumed by *Oreochromis niloticus* from Sor River using Frequency of occurrence (% F), Numerical index (% N) and gravimetric (% G) index values analysis.

The stomach content analysis of Nile tilapia (*Oreochromis niloticus*) from Sor River revealed phytoplankton, macrophytes, insects, debris and sand (Table 2). The present study also indicated phytoplankton, macrophytes and debris were the most important food constituent of *O. niloticus* diet in Sor River (Fig 2).

**Table 2:** Index values and corresponding Index of Relative importance (% IRI) of the various food items in the diets of *O. niloticus from* Sor River.

Food items	% F	% N	% G	IRI	% IRI
Phytoplankton	85.71	37.82	31.03	3833.94	39.85
Macrophytes	71.43	21.24	24.14	2236.91	23.25
Insects	28.57	5.44	8.62	293.21	3.05
Detritus	85.71	18.91	20.69	2164.68	22.50
Sand grains	42.86	7.77	5.17	261.87	2.72
Unidentified	71.43	8.81	10.34	830.04	8.63

### Labeo forskalii

**Fig 3:**
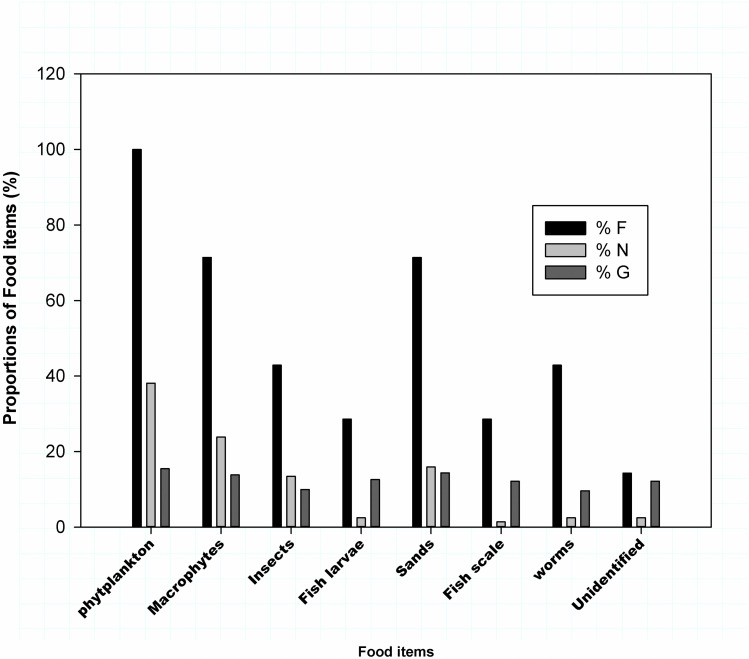
The percentage contribution of food items consumed by *Labeo forskalii* from Sor River using Frequency of occurrence (% F), Numerical index (% N) and gravimetric (% G) index values analysis.

The stomach content analysis showed *Labeo forskalii feeds* on Phytoplankton, macrophytes, insects, fish larvae, Detritus and others (Table 3). Index of relative importance analysis indicted phytoplankton, macrophytes, insects, and Detritus are the most important preys (Fig 3).

**Table 3:** Index of Relative importance (%IRI) values of the various food items in the diets of *Labeo forskalii* from Sor River.

Food Items	% F	% N	% G	IRI	% IRI
Phytoplankton	100.00	38.08	15.45	2133.72	32.07
Macrophytes	71.43	23.84	13.80	1314.35	19.76
Insects	42.86	13.42	9.93	559.09	8.40
Fish larvae	28.57	2.47	12.58	390.53	5.87
Detritus & sand	71.43	15.89	14.35	1252.92	18.83
Fish scale	28.57	1.37	12.14	363.53	5.46
Flat worms	42.86	2.47	9.60	435.22	6.54
Unidentified	14.29	2.47	12.14	203.38	3.06

### Labeobarbus intermedius

**Fig 4:**
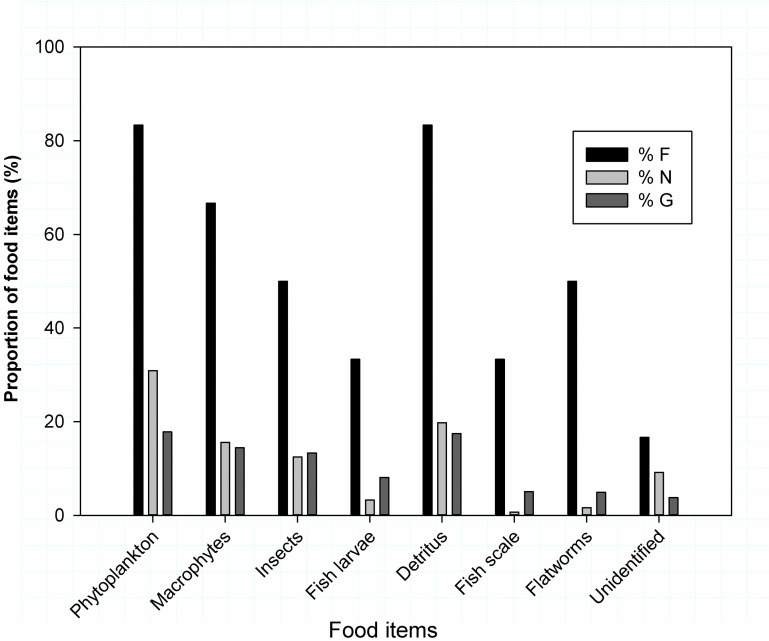
The percentage contribution of food items consumed by Labeobarbus intermedius from Sor River using Frequency of occurrence (% F), numerical (% N) and gravimetric (% G) index value analysis.

The gut content analysis *L. intermedius* from Sor River revealed phytoplankton and detritus were the dominant food items (Table 4). Macrophytes, gastropods, insects, fish larvae, fish scale and flatworms are also important diet composition (Fig 4).

**Table 4:** Index of Relative importance (%IRI) values of the various food items in the diets of *Labeobarbus intermedius* from Sor River.

Food items	% F	% N	% G	IRI	% IRI
Phytoplankton	83.33	30.90	17.81	2033.98	23.72
Macrophytes	66.67	15.57	14.47	1189.69	13.87
Insects	50.00	12.50	13.35	1057.23	12.33
Fish larvae	33.33	3.30	8.11	432.18	5.04
Detritus and sand	83.33	19.81	17.49	1803.80	21.04
Fish scale	33.33	0.71	5.09	173.18	2.02
Flatworms	50.00	1.65	4.93	254.56	2.97
Unidentified	16.67	9.20	3.82	289.47	3.38

## Discussion

In terms of prey importance, the foods of plant origin (mainly phytoplankton) are the most consumed food types by the Fish. The stomach content analysis of *O. niloticus* has shown that it feeds on a phytoplankton, macrophytes and debris as the main food constituent in Sor River. The results observed in this study as well as previous studies confirm that *O*. *niloticus* is omnivorous [17–21]. Research conducted in a few Ethiopian rift valley lakes, including Lake Hawassa [22], Lake Chamo [23], Lake Ziway [17], Lake Langeno [21], and Koka Reservoir ([18]); a few highland lakes, such as Lake Hayq [24], in the lower Omo basin, Gilgel Gibe Reservoir [19] and River Omo [25], and Tekeze basin ([20]), revealed that phytoplankton is the primary food source for *O. niloticus*, which is consistent with the current study conducted in the Sor River.

*Labeo forskalii* was previously reported in the IUCN Red List of threatened species [26]. However, Melaku *et al.* [2] reported the existence of *L. forskalii* from Sor and Gebba Rivers in population aspects of Fish. Teshome *et al.* [27] confirmed the existence of *L. forskalii* from upper Blue Nile River and mainly feeds on mud and Phytoplankton as major food constituent. The current study also revealed, *Labeo forskalii feeds* on Phytoplankton, macrophytes, insects, fish larvae, and detritus.

The feeding habit of *L. intermedius* from different water bodies of Ethiopia indicate that the fish is omnivorous and mainly feeds on gastropods, phytoplankton, insects, insect larvae, nematodes, macrophytes, Detritus and others [28]. Study from Lake Koka by Tesfahun [29] reveals the piscivorous feeding habit of the fish where the gut contents were found to be composed of fish, fish scales, fish fry, fish egg and other common insects and nematodes. Study from Lake Tana, Engdaw [30] reported detritus were the most important food that dominates the gut content of *L. intermedius*. Insects, phytoplankton and gastropods were also important food items in the diet of *L. intermedius.* The gut content analysis *L. intermedius* from Sor River revealed phytoplankton and detritus were the dominant food items. Macrophytes, insects, fish larvae, fish scale and flatworms are also important diet composition.

The diet breadth index (B_A_), which measures the diversity of prey items consumed by each fish species, was 0.43 for *O. niloticus,* 0.61 for *L. intermedius and* 0.64 for *Labeo forskalii,* respectively. Horn’s (H) diet overlap index was 0.46 for *L forskalii, L. intermedius and O. niloticus*. According to their diet breadth and overlap indices, the three fish species had an omnivorous feeding habit, taking advantage of the river’s rich food resources.

## Conclusions

The diet composition and feeding habit of *Oreochromis niloticus*, *Labeo forskalii and Labeobarbus intermedius* were examined*. Oreochromis niloticus* from Sor River feed on phytoplankton, detritus and macrophtes as major prey. *Labeo forskalii stomach consists of*, insects, fish larvae, Detritus in addition to *Phytoplankton*, macrophytes while *Labeobarbus intermedius* from Sor mainly feeds phytoplankton and detritus. According to their diet breadth and overlap indices, the three fish species had an omnivorous feeding habit which is useful to enhance fisheries and aquaculture development in the study area*.* However, the biology and behaviour of most species in Sor River is still lacking. As a result, additional research on the biology and behaviour of fish in the study area is required. It is also recommendable to further investigate the impact of environmental factors and seasonal variations on fish feeding behaviour.

## Supporting Information

S1 Table *Labeo forskalii* TL (cm), BW (gm) and gut constituent (gm) (docx).
(DOCX)

S2 Table *Labeobarbus intermedius* TL (cm), BW (gm) and gut constituent (gm) (docx).
(DOCX)

S3 Table *Oreochromis niloticus* TL (cm), BW (gm) and gut constituent (gm) (docx).
(DOCX)

S4 Table Gut constituent of *Labeo forskalii, Labeobarbus intermedius* and *Oreochromis niloticus* from Sor River (docx).
(DOCX)

## References

[1] Abegaz H, Tesfaye G, Cheffo A. Fishery development program: riverine fishery assessment in gambella peoples’ regional state. Int J Fish Aquac. 2010;11(1):7–12.

[2] Melaku S, Getahun A, Wakjira M. Population aspects of fish in Geba and Sor rivers, White Nile system in Ethiopia, East Africa. Int J Biodivers. 2017;125.

[3] Tesso TA, Melaku S, Dobamo T. Assessing Fishing activity, Fish Production and demand outlook in Ilu Abba Bora Zone, Oromia Regional State, South West Ethiopia. GJAS. 2017;7(1):009–18. doi: 10.15580/gjas.2017.1.120316208

[4] Dadebo E, Mengistou S. Feeding habits, ontogenetic dietary shift and some aspects of reproduction of the tigerfish Hydrocynus forskahlii (Cuvier 1819) (Pisces: Characidae) in Lake Chamo, Ethiopia. Ethiop J Biol Sci. 2008;7(2):123–37.

[5] Shalloof KA, Khalifa N. Stomach contents and feeding habits of Oreochromis niloticus (L.) from Abu-Zabal lakes, Egypt. World Appl Sci J. 2009;6(1):1–5.

[6] Adeyemi S, Bankole N, Adikwu I, Akombu P. Age, growth and mortality of some commercially important fish species in Gbedikere Lake, Kogi State Nigeria. Int J Lakes Rivers. 2009;2(1):45–51.

[7] Otieno O, Kitaka N, Njiru J. Length-weight relationship, condition factor, length at first maturity and sex ratio of Nile tilapia, Oreochromis niloticus in Lake Naivasha, Kenya. Int J Fish Aquat Stud. 2014;2(2):67–72.

[8] Philips M, Carillet J. Ethiopia and Eritrea, third edition, n.p.: Lonely Planet, 2006.p. 240.

[9] Willen E. A simplified method of phytoplankton counting. Br Phycol J. 1976;11(3):265–78.

[10] Bowen S. Quantitative description of the diet. Fisheries Techniques, 2nd edition. Bethesda, Maryland: American Fisheries Society. 1996:513–32.

[11] Whitford LA. Additions to the freshwater algae in North Carolina. IX. J Elisha Mitchell Sci Soc. 1979;95(1):42–7.

[12] Defaye D. A new Gaussia (Copepoda, Calanoida, Metridinidae) from the North Pacific. Crustaceana. 1998;71(1):81–91.

[13] Pennak RW. Fresh-water invertebrates of the United States.1978, 2nd ed. John Wiley & Sons.

[14] Windell J, Bowen S. Methods for study of fish diets based on analysis of stomach contents. IBP Handbook. 1978.

[15] Hynes HBN. The Food of Fresh-Water Sticklebacks (Gasterosteus aculeatus and Pygosteus pungitius), with a Review of Methods Used in Studies of the Food of Fishes. J Anim Ecol. 1950;19(1):36. doi: 10.2307/1570

[16] Gelwick FP, Matthews WJ. Trophic relations of stream fish 2007. In: Hauer FR, Lamberti GA (eds) Methods in Stream Ecology. 2nd ed. pp 611–636, Amsterdam: Elsevier Inc.

[17] Negassa A, Prabu P. Abundance, food habits, and breeding season of exotic Tilapia zillii and native Oreochromis niloticus L. fish species in Lake Zwai, Ethiopia. Maejo Int J Sci Technol. 2008;2(2):345–59.

[18] Engdaw F, Dadebo E, Nagappan R. Morphometric relationships and feeding habits of Nile tilapia Oreochromis niloticus (L.) (Pisces: Cichlidae) from Lake Koka, Ethiopia. Int J Fish Aquat Sci. 2013;2(4):65–71.

[19] Wakjira M. Feeding habits and some biological aspects of fish species in Gilgel Gibe Reservoir, Ethiopia. Int J Curr Res. 2013;5(12):4124–32.

[20] Teame T, Natarajan P, Tesfay Z. Assessment of fishery activities for enhanced management and improved fish production in Tekeze reservoir, Ethiopia. Int J Fauna Biol Stud. 2016;3(1):105–13.

[21] Temesgen M. Status and trends of fish and fisheries in Lake Langano, Ethiopia (Doctoral dissertation, PhD dissertation, Addis Ababa University, Ethiopia. p. 231).

[22] Tadesse Z. The nutritional status and digestibility of Oreochromis niloticus L. diet in Lake Langeno, Ethiopia. Hydrobiology. 1999;416:976–106.

[23] Teferi Y, Admassu D, Mengistou S. The food and feeding habit of <i>Oreochromis niloticus</i> L. (Pisces: Cichlidae) in Lake Chamo, Ethiopia. SEJS. 2000;23(1):. doi: 10.4314/sinet.v23i1.18152

[24] Workiyie W, Abebe G. The food and feeding ecology of Nile tilapia, Oreochromis niloticus, in Lake Hayq, Ethiopia. Int J Ecol Environ Sci. 2015;41(1/2):55–65.

[25] Wakjira M. Fish diversity, community structure, feeding ecology, and fisheries of lower Omo River and the Ethiopian part of Lake Turkana, East Africa. (Doctoral dissertation, Addis Ababa University), 2016.

[26] Getahun A, Twongo T. Labeo forskalii. The IUCN Red List of Threatened Species. 2010. doi: 10.2305/IUCN.UK.20103.RLTS.T181762A7727627.en

[27] Dadebo E, Tesfahun A, Teklegiorgis Y. Food and feeding habits of the African big barb Labeobarbus intermedius (Rüppell, 1836) (Pisces: Cyprinidae) in Lake Koka, Ethiopia. E3 J Agric Res Dev. 2013;3(4):49–58.

[28] Tesfahun A. Morphometric relationships and feeding habits of the African big barb, Barbus intermedius (Rüpell, 1836) (Pisces: Cyprinidae): in Lake Koka, Ethiopia (Doctoral dissertation), 2011.

[29] Engdaw F. Morphometric relations, diet composition and ontogenetic dietary shift of Labeobarbus intermedius (Rppell, 1836) in Lake Tana gulf of Gorgora, Ethiopia. Int J Fish Aquac. 2014;6(11):124–32.

