# Correction: Feeding habit and diet composition of three fish species inhabiting Sor River, Baro-Akobo Basin of Ethiopia, East Africa

**DOI:** 10.1371/journal.pone.0325035

**Published:** 2025-05-21

**Authors:** Tujuba Ayele Tesso, Limatu Kebede Nurgi

There are errors in the Author Contributions. The correct contributions are:

**Conceptualization:** Tujuba Ayele Tesso, Limatu Kebede Nurgi.

**Data Curation:** Tujuba Ayele Tesso, Limatu Kebede Nurgi.

**Funding acquisition:** Tujuba Ayele Tesso.

**Formal Analysis:** Tujuba Ayele Tesso, Limatu Kebede Nurgi.

**Investigation:** Tujuba Ayele Tesso, Limatu Kebede Nurgi.

**Methodology:** Tujuba Ayele Tesso, Limatu Kebede Nurgi.

**Project administration:** Tujuba Ayele Tesso.

**Software:** Tujuba Ayele Tesso, Limatu Kebede Nurgi.

**Supervision:** Tujuba Ayele Tesso.

**Visualization:** Tujuba Ayele Tesso.

**Writing – original draft:** Tujuba Ayele Tesso.

**Writing – review & editing:** Tujuba Ayele Tesso, Limatu Kebede Nurgi.
