# Correction: Correction: Feeding habit and diet composition of three fish species inhabiting Sor River, Baro-Akobo Basin of Ethiopia, East Africa

**DOI:** 10.1371/journal.pone.0328973

**Published:** 2025-07-23

**Authors:** Tujuba Ayele Tesso, Limatu Kebede Nurgi

There are errors in the Correction published on May 21, 2025. The errors are in the Author Contributions. The correct contributions are:

**Conceptualization**: Tujuba Ayele Tesso and Limatu Kebede Nurgi.

**Data Curation**: Tujuba Ayele Tesso and Limatu Kebede Nurgi.

**Funding acquisition**: Tujuba Ayele Tesso and Limatu Kebede Nurgi.

**Formal Analysis**: Tujuba Ayele Tesso and Limatu Kebede Nurgi.

**Investigation**: Tujuba Ayele Tesso and Limatu Kebede Nurgi.

**Methodology**: Tujuba Ayele Tesso and Limatu Kebede Nurgi.

**Project administration**: Tujuba Ayele Tesso and Limatu Kebede Nurgi.

**Software**: Tujuba Ayele Tesso and Limatu Kebede Nurgi.

**Supervision**: Tujuba Ayele Tesso.

**Visualization**: Tujuba Ayele Tesso.

**Writing – original draft**: Tujuba Ayele Tesso.

**Writing – review & editing**: Tujuba Ayele Tesso and Limatu Kebede Nurgi.

## References

[1] Tesso TA, Nurgi LK. Feeding habit and diet composition of three fish species inhabiting Sor River, Baro-Akobo Basin of Ethiopia, East Africa. PLoS One. 2025;20(3):e0319927. doi: 10.1371/journal.pone.0319927 40117287 PMC11927877

[2] Tesso TA, Nurgi LK. Correction: Feeding habit and diet composition of three fish species inhabiting Sor River, Baro-Akobo Basin of Ethiopia, East Africa. PLoS One. 2025;20(5):e0325035. doi: 10.1371/journal.pone.0325035 40117287 PMC11927877

