# Supplementary material for: Feeding habit and diet composition of three fish species inhabiting Sor River, Baro-Akobo Basin of Ethiopia, East Africa
Source: PLoS One. 2025 Mar 21;20(3):e0319927. doi: 10.1371/journal.pone.0319927 (PMC11927877; doi:10.1371/journal.pone.0319927)
Supplement: S1 Table — (DOCX) [file pone.0319927.s001.docx]

**S1 Table. *Labeo forskali* TL (cm), BW (gm) and gut constituent (gm)**

| Sample no. | Fish species | TL (cm) | BW (gm) | Gut content (gm) |
| --- | --- | --- | --- | --- |
| S1 | *Labeo forskalii* | 34 | 650 | 120.02 |
| S2 | *Labeo forskalii* | 36 | 700 | 210.53 |
| S3 | *Labeo forskalii* | 34 | 500 | 110.84 |
| S4 | *Labeo forskalii* | 30 | 490 | 105.66 |
| S5 | *Labeo forskalii* | 28 | 400 | 102.55 |
| S6 | *Labeo forskalii* | 26 | 350 | 90.32 |
| S7 | *Labeo forskalii* | 23 | 420 | 90.12 |
| S8 | *Labeo forskalii* | 21 | 335 | 95.32 |
| S9 | *Labeo forskalii* | 18 | 240 | 87.62 |
| S10 | *Labeo forskalii* | 21 | 290 | 78.23 |
| S11 | *Labeo forskalii* | 30 | 234 | 45.60 |
| S12 | *Labeo forskalii* | 29 | 250 | 46.34 |
| S13 | *Labeo forskalii* | 22 | 520 | 104.52 |
| S14 | *Labeo forskalii* | 23 | 430 | 96.50 |
