# Supplementary material for: Feeding habit and diet composition of three fish species inhabiting Sor River, Baro-Akobo Basin of Ethiopia, East Africa
Source: PLoS One. 2025 Mar 21;20(3):e0319927. doi: 10.1371/journal.pone.0319927 (PMC11927877; doi:10.1371/journal.pone.0319927)
Supplement: S2 Table — (DOCX) [file pone.0319927.s002.docx]

**S2 Table. *Labeobarbus intermidus* TL (cm), BW (gm) and gut constituent (gm)**

| Sample no. | Fish species | TL (cm) | BW (gm) | Gut content (gm) |
| --- | --- | --- | --- | --- |
| S1 | *Labeobarbus intermidus* | 42 | 650 | 130.85 |
| S2 | *Labeobarbus intermidus* | 36 | 470 | 118.31 |
| S3 | *Labeobarbus intermidus* | 38 | 590 | 126.33 |
| S4 | *Labeobarbus intermidus* | 36 | 520 | 116.34 |
| S5 | *Labeobarbus intermidus* | 24 | 360 | 91 |
| S6 | *Labeobarbus intermidus* | 26 | 370 | 67 |
| S7 | *Labeobarbus intermidus* | 28 | 380 | 68 |
| S8 | *Labeobarbus intermidus* | 23 | 255 | 48 |
| S9 | *Labeobarbus intermidus* | 18 | 170 | 23 |
| S10 | *Labeobarbus intermidus* | 19 | 180 | 18 |
| S11 | *Labeobarbus intermidus* | 24 | 360 | 89 |
| S12 | *Labeobarbus intermidus* | 27 | 390 | 79 |
| S13 | *Labeobarbus intermidus* | 22 | 290 | 67 |
| S14 | *Labeobarbus intermidus* | 23 | 255 | 14 |
| S15 | *Labeobarbus intermidus* | 35 | 458 | 102 |
| S16 | *Labeobarbus intermidus* | 34 | 410 | 87 |
| S17 | *Labeobarbus intermidus* | 41 | 600 | 128 |
| S18 | *Labeobarbus intermidus* | 42 | 620 | 124 |
| S19 | *Labeobarbus intermidus* | 23 | 260 | 23 |
| S20 | *Labeobarbus intermidus* | 34 | 450 | 76 |
| S21 | *Labeobarbus intermidus* | 35 | 460 | 82 |
| S22 | *Labeobarbus intermidus* | 36 | 470 | 91 |
| S23 | *Labeobarbus intermidus* | 24 | 270 | 25 |
| S24 | *Labeobarbus intermidus* | 21 | 290 | 23 |
| S25 | *Labeobarbus intermidus* | 19 | 190 | 18 |
| S26 | *Labeobarbus intermidus* | 31 | 395 | 34 |
| S27 | *Labeobarbus intermidus* | 29 | 390 | 46 |
| S28 | *Labeobarbus intermidus* | 25 | 370 | 38 |
