# Supplementary material for: Feeding habit and diet composition of three fish species inhabiting Sor River, Baro-Akobo Basin of Ethiopia, East Africa
Source: PLoS One. 2025 Mar 21;20(3):e0319927. doi: 10.1371/journal.pone.0319927 (PMC11927877; doi:10.1371/journal.pone.0319927)
Supplement: S4 Table — (DOCX) [file pone.0319927.s004.docx]

**S4 Table. Gut constituent of** *Labeo forskalii****,*** *Labeobarbus intermidus* **and** *Oriochrmois niloticus* **from Sor River**

|  | | Feed type | S1 | S2 | S3 | S4 | S5 | Total count |
| --- | --- | --- | --- | --- | --- | --- | --- | --- |
| *Labeo forskalii* |  | Phytoplankton | 42 | 26 | 0 | 50 | 21 | 139 |
|  |  | Macrophytes | 35 | 40 | 0 | 12 | 0 | 87 |
|  |  | Insects | 12 | 0 | 8 | 18 | 11 | 49 |
|  |  | fish larvae | 0 | 3 | 0 | 0 | 6 | 9 |
|  |  | detritus & sand | 20 | 13 | 14 | 7 | 4 | 58 |
|  |  | fish scale | 2 | 0 | 0 | 0 | 3 | 5 |
|  |  | flat worms | 4 | 2 | 0 | 0 | 3 | 9 |
|  |  | unidentified | 0 | 0 | 0 | 9 | 0 | 9 |
|  |  | **Total** |  |  |  |  |  | **365** |
| *Labeobarbus intermidus* | | Phytoplankton | 21 | 43 | 51 | 16 | 0 | 131 |
|  |  | macrophytes | 12 | 10 | 9 | 35 | 0 | 66 |
|  |  | Insects | 13 | 5 | 14 | 21 | 0 | 53 |
|  |  | fish larvae | 2 | 5 | 0 | 7 | 0 | 14 |
|  |  | detritus | 25 | 14 | 11 | 34 | 0 | 84 |
|  |  | sand grains | 12 | 5 | 8 | 2 | 0 | 27 |
|  |  | fish scale | 2 | 0 | 1 | 0 | 0 | 3 |
|  |  | flat worms | 0 | 2 | 1 | 4 | 0 | 7 |
|  |  | unidentified | 10 | 4 | 5 | 20 |  | 39 |
|  |  | **Total** |  |  |  |  |  | **424** |
|  |  |  |  |  |  |  |  |  |
| *Oriochrmois niloticus* | | Phytoplankton | 61 | 30 | 55 | 0 | 0 | 146 |
|  |  | macrophytes | 3 | 28 | 51 | 0 | 0 | 82 |
|  |  | Insects | 15 | 6 | 0 | 0 | 0 | 21 |
|  |  | detritus | 21 | 18 | 34 | 0 | 0 | 73 |
|  |  | sand grains | 12 | 18 | 0 | 0 | 0 | 30 |
|  |  | unidentified | 14 | 11 | 9 | 0 | 0 | 34 |
|  |  | **Total** |  |  |  |  |  | **386** |
